# Clinical Approach to Pediatric Transverse Myelitis, Neuromyelitis Optica Spectrum Disorder and Acute Flaccid Myelitis

**DOI:** 10.3390/children6050070

**Published:** 2019-05-17

**Authors:** Cynthia Wang, Benjamin Greenberg

**Affiliations:** 1 Department of Pediatrics, University of Texas Southwestern Medical Center, Dallas, TX 75390, USA; Benjamin.greenberg@utsouthwestern.edu; 2 Department of Neurology and Neurotherapeutics, University of Texas Southwestern Medical Center, Dallas, TX 75390, USA

**Keywords:** transverse myelitis, neuromyelitis optica spectrum order, acute flaccid myelitis, pediatric, review

## Abstract

Pediatric transverse myelitis (TM) is an acquired, immune-mediated disorder that leads to injury of the spinal cord and often manifests as weakness, numbness, bowel dysfunction, and/or bladder dysfunction. Multiple etiologies for myelitis can result in a similar clinical presentation, including idiopathic transverse myelitis (TM), multiple sclerosis (MS), neuromyeltis optica spectrum disorder (NMOSD) associated with anti-aquaporin 4 antibodies, MOG antibody-associated disease, and acute flaccid myelitis (AFM). Diagnosis relies on clinical recognition of the syndrome and confirming inflammation through imaging and/or laboratory studies. Acute treatment is targeted at decreasing immune-mediated injury, and chronic preventative therapy may be indicated if TM is determined to be a manifestation of a relapsing disorder (i.e., NMOSD). Timely recognition and treatment of acute transverse myelitis is essential, as it can be associated with significant morbidity and long-term disability.

## 1. Introduction

Pediatric transverse myelitis (TM) accounts for about 20% of the total number of cases of transverse myelitis [1]. Transverse myelitis can result from multiple pathological mechanisms. Idiopathic transverse myelitis refers to a single episode of TM, postulated to arise from post-infectious immune activation. TM can also be a part of a relapsing inflammatory disorder, such as multiple sclerosis (MS) or neuromyelitis optica spectrum disorder (NMOSD). Infections of the spinal cord cause direct tissue injury, and in some cases, may trigger downstream immune mechanisms that lead to additional injury (i.e., acute flaccid myelitis).

## 2. Demographic Features and Epidemiology

The incidence of acute transverse myelitis (ATM) in children under the age of 16 years is estimated to be 2 per million children per year based on studies in the UK and Canada [2]. Within the pediatric population, idiopathic myelitis has been more frequently reported in children under 5 and over 10 years of age [3]. There is a slight male predominance (1.1–1.6:1) in prepubertal individuals, while female predominance is observed following puberty, particularly in cases in which TM represents a manifestation of MS or NMOSD. No ethnic predisposition has been reported. A preceding illness is noted in two-thirds of cases [4].

## 3. Clinical Features

Clinical manifestations of TM may include pain, paresthesias, numbness, weakness (typically paraplegia or quadriplegia), bowel dysfunction, and/or bladder dysfunction. During the acute setting, muscle tone and deep tendon reflexes in affected extremities may be decreased, but typically increased tone and hyperreflexia (secondary to corticospinal tract involvement) becomes apparent over time. The time course from symptom onset to maximal severity can vary considerably depending on the etiology and severity of disease, ranging from 4 hours to 21 days [5]. The diagnostic criteria established by the Transverse Myelitis Consortium Working Group (TMCWG), which includes bilateral extremity weakness and a clear sensory level, can be utilized in children with the caveat that younger children may not reliably report a sensory level.

## 4. Diagnostic Approach

A diagnosis of transverse myelitis can be established with a consistent clinical history and neurological exam, supported by radiographical and laboratory studies, and exclusion of alternate etiologies. An acute to subacute time course is typical of transverse myelitis given its immune-mediated mechanism. Hyperacute or chronic development of symptoms prompt consideration of alternative etiologies, such as vascular and metabolic myelopathies, respectively. TM can be mistaken for peripheral nervous system disorders, such as Guillain–Barré syndrome (GBS), as both can acutely manifest as weakness and areflexia. However, a sensory level and urinary retention should raise concern for spinal cord localization. Furthermore, a variant of TM, acute flaccid myelitis (AFM) can mimic axonal GBS, but causes changes in the anterior horns of the spinal cord when magnetic resonance imaging (MRI) imaging is obtained. Once there is clinical suspicion for myelopathy, it should be treated as a medical emergency, as a compressive lesion must be ruled out. Cerebrospinal fluid (CSF) studies are also used to evaluate for infectious myelitis, but should not delay initiation of empirical intravenous corticosteroids if there is suspicion for an inflammatory myelopathy.

## 5. Diagnostic Workup

### 5.1. Magnetic Resonance Imaging (MRI)

Magnetic resonance imaging (MRI) of the spinal cord should be obtained to evaluate for extrinsic and intrinsic causes of myelopathy. Transverse myelitis typically manifests as abnormal T2/fluid-attenuated inversion recovery (FLAIR) hyperintensities affecting one or more cord segments. Shorter segment lesions are more suggestive of multiple sclerosis-related TM, while lesions longer than three vertebral segments increase the probability of NMOSD and idiopathic TM. Children are more likely to have longitudinally extensive myelitis regardless of etiology, thus making the length of the lesion less diagnostically useful in this population. Enhancement can be present but is not necessary to diagnose TM. Idiopathic transverse myelitis and NMOSD-associated myelitis often manifest as central lesions that can encompass two-thirds or more of the cross section of the cord [6]. In contrast, acute flaccid myelitis has predominantly gray matter involvement, and mixed patterns have been observed in MOG-antibody associated myelitis [7,8].

### 5.2. Cerebrospinal Fluid (CSF)

Cerebrospinal fluid analysis should include evaluation of cell count and differential, protein, and glucose analysis. The CSF is abnormal in about 50% of TM cases, often with lymphocytic pleocytosis, elevated protein, but normal glucose. Children with myelitis tend to have a pleocytosis of greater magnitude than adults, with a mean white blood cell count of 136/mm^3^ [1]. Evidence of intrathecal antibody synthesis, as demonstrated by positive oligoclonal bands and increased Immunoglobulin G (IgG) index, are suggestive of autoimmune myelitis. This pattern is observed most commonly with multiple sclerosis, although other autoimmune as well as infectious etiologies can also produce such findings. Aquaporin-4 antibody can be assessed in CSF, though the serum test is more sensitive. Assessing for viral pathogens, such as enterovirus, West Nile virus, and other arboviruses associated with myelitis, may be relevant depending on the time of year and geographical location. Of note, serologic testing or viral isolation from non-central nervous system (CNS) sources is often necessary to prove viral pathology in the setting of myelitis.

### 5.3. Serum

Serum laboratory testing for anti-AQP4 and MOG antibodies through cell-based assays should be considered in any child with myelitis, as a positive result would be clinically significant in relation to ongoing surveillance and management. Paraneoplastic myelitis is rare and sometimes associated with amphiphysin and CRMP-5 antibodies in adults [9,10], but has not been reported in children. Anti-GFAP syndromes have been described in pediatric patients and can manifest as meningoencephalomyelitis with spinal cord lesions spanning greater than three vertebral segments. A distinguishing MRI brain feature is a radial enhancement pattern [11]. Evaluation for metabolic myelopathies with testing of vitamin B12, copper, and vitamin E should be considered in children at risk for gastrointestinal and metabolic comorbidities. 25-Hydroxy vitamin D level is associated with a greater risk of acquiring MS and having more active disease. It may be important in other autoimmune neurological disorders as well and should be evaluated to guide dosing recommendations.

Infectious serologies for West Nile virus, varicella-zoster virus, herpes simplex virus, human T lymphotropic virus, human immunodeficiency virus (HIV), and Zika virus are additional tests that might be considered in the appropriate clinical context. Bacterial infections including mycoplasma, *Borrelia* species, syphilis, and *Listeria* monocytogenes have also been reported in association with myelitis.

### 5.4. Other Studies

Additional studies can be considered based on the patient’s history and examination. If there is concern for sarcoidosis, computerized tomography (CT) of chest can help screen for granulomatous lung disease, although this is rare in children. Paraneoplastic workup might involve CT chest, abdomen, and pelvis, though these studies typically have very low yield in children. Electrophysiological and other adjunctive tests, including visual evoked potentials, nerve conduction studies/electromyography and optical coherence tomography to identify patterns of diffuse CNS inflammation, may be helpful if the etiology of myelitis is uncertain.

## 6. Acute Treatment

Acute management of transverse myelitis has been informed primarily by case series and expert opinion, as no randomized controlled trials have been done in this population. The American Academy of Neurology (AAN) guidelines states that corticosteroids only have class IV evidence to support their use, though in practice, they are often first-line treatments and have low risk for potential harm even in cases of transverse myelitis mimics. Typically, corticosteroids are given as 30 mg/kg (up to 1000 mg) of intravenous methylprednisolone daily for 3–5 days. Other preparations of corticosteroids, such as dexamethasone or oral administration of high-dose prednisone or prednisolone, may also be used.

With severe cases of myelitis or symptoms refractory to IV corticosteroids, therapeutic plasma exchange (PLEX) has been a very effective acute treatment for acute transverse myelitis, particularly in conditions in which a pathogenic antibody is felt to be central to the disease process. Plasma exchange has been shown to be effective in idiopathic transverse myelitis, neuromyelitis optica, and MOG antibody associated disease. Studies of plasma exchange in children have demonstrated that it is a safe and effective treatment [12]. The typical treatment regimen involves 1.1–1.5 plasma volume changes every other day for 5–7 sessions.

Other frequently used acute therapies include intravenous immunoglobulin (IVIG), which can also be given concurrently or following IV corticosteroids. Certain forms of myelitis, particularly those related to systemic inflammatory and connective tissue disorders, such as systemic lupus erythematosus, may respond preferentially to treatments such as cyclophosphamide [13].

## 7. Forms of Acute Myelitis

Idiopathic transverse myelitis is often diagnosed when no specific etiology is found following an appropriate and comprehensive workup. In many cases, there is a history of a preceding respiratory or gastrointestinal illness. These forms of TM are often monophasic, but can cause significant morbidity. With improvements in molecular testing techniques, some of these conditions are now known to represent forms of neuromyelitis optica spectrum disorder, MOG antibody associated disease, and multiple sclerosis. In one study of adults previously diagnosed with idiopathic TM, an alternative and specific myelopathy diagnosis was made in 69.9% of 226 referred patients, which was led by vascular myelopathy and clinically isolated syndrome/multiple sclerosis [14].

### 7.1. Neuromyelitis Optica Spectrum Disorders (NMOSD)

Neuromyelitis optica spectrum disorder (NMOSD)-related myelitis is classically described to be longitudinally extensive, involving three or more vertebral segments. It is frequently associated with optic neuritis and typically involves severe attacks without disease progression between relapses. The discovery of its antigenic target, aquaporin-4, and improvements in cell-based assays have broadened our understanding of the spectrum of neuromyelitis optica. Shorter NMOSD lesions have been reported [15] and brainstem, diencephalon, and brain involvement have also been increasingly recognized. Younger children tend to have longitudinally extensive myelitis, such as with MOG-antibody associated myelitis and idiopathic TM, so this characteristic of anti-AQP4 antibody-mediated NMOSD is less specific in the pediatric population. Natural history studies have demonstrated that if untreated, NMOSD leads to high morbidity and mortality, with over 50% of patients with relapsing neuromyelitis optica becoming blind in one or both eyes or requiring ambulatory help within 5 years of disease onset [16]. Thus, all patients with acute transverse myelitis should be tested for anti-AQP4 antibodies.

Therapeutic plasma exchange for severe presentations and/or incomplete recovery following corticosteroids is recommended, as it often improves motor and vision outcomes [17]. Chronic immunosuppression should be initiated after the first attack in individuals positive for AQP-4 and a compatible clinical history, as poor recovery from exacerbations greatly contributes to disease-associated disability.

### 7.2. Acute Flaccid Myelitis (AFM)

Acute flaccid myelitis (AFM) has been an increasingly recognized syndrome in children. In this condition, typically children or young adults present with rapidly progressive, often asymmetric, flaccid weakness. Over 90% of affected individuals have a mild viral respiratory illness or fever before the onset of neurological symptoms [18]. Areflexia or hyporeflexia is present in the most severely affected limbs, though reflexes can be normal or brisk below the level of spinal cord injury. Sensation and bowel and bladder function can be relatively spared. Radiographically, AFM corresponds to hyperintense T2 signal in the central gray matter of the spinal cord, particularly the anterior horns.

The clinical and radiological phenotype of AFM is similar to poliomyelitis. Although the cause of acute flaccid myelitis (AFM) is still unproven, a temporal and geographical correlation with enterovirus infections has led to this being the most favored etiology. The most frequently associated virus has been the enterovirus D68, being detected in respiratory specimens, but CSF testing is almost always negative for enterovirus when tested with PCR. Enterovirus A71 has been implicated in other cohorts of AFM patients [19]. The Center for Disease Control and Prevention (CDC)’s case classification for AFM requires an MRI showing spinal cord lesion largely restricted to gray matter and spanning one or more spinal segments in a clinically compatible case. The majority of established cases have occurred in the fall seasons of 2014 (120 cases), 2016 (149 cases), and 2018 (223 cases). Prospective epidemiological studies are needed to establish a causal relationship between AFM and specific enterovirus subtypes and determine what makes certain individuals more susceptible to developing AFM.

Presently, the CDC states that there are currently no targeted therapies/interventions with enough evidence to endorse or discourage their use for the treatment or management of AFM, including corticosteroids, IVIG, and therapeutic exchange. Other treatments that have been proposed include fluoxetine, antiviral medications, interferon, and other immunosuppressive medications, but these are not recommended by the CDC.

## 8. Prognosis/Long-Term Management

Although many children have substantial recoveries from transverse myelitis, as high as 40% have residual deficits that interfere with activities of daily living and impact quality of life [1]. Improvement in spinal cord function is typically most apparent in the first three months following the onset of symptoms, but can continue over many years with appropriate rehabilitation. Motor recovery from AFM is slower and less complete compared to immune-mediated myelitis. In the 2014 Colorado cohort, the majority of children exhibited persistent motor deficits at 1 year [20].

Recurrence of TM is very unlikely in children with myelitis due to an identifiable viral etiology and no child with AFM has been reported to have subsequent myelitis events.

For children determined to have myelitis secondary to autoimmune condition, chronic immunomodulatory therapy should be considered. Since NMOSD has a well characterized relapsing disease course, identification of AQP4 antibodies and a clinical attack meeting Wingerchuk diagnostic criteria for NMOSD should prompt initiation of immunosuppression. 

Presently, rituximab, mycophenolate mofetil, azathioprine, and daily corticosteroids are the most frequently utilized treatments based on expert opinion and retrospective studies. Several therapies of differing mechanisms have completed phase III clinical trials in adults with NMOSD.

## 9. Summary/Future Directions 

Our understanding of pediatric TM has grown significantly with the discoveries of antibody-mediated forms of myelitis and the identification of new potential infectious etiologies. Despite differences in pathobiology, the clinical presentation of myelitis shares many features. Effective management of myelitis requires recognition of the syndrome, initiating empirical treatment, and using imaging and laboratory studies to establish a cause and guide medical decision-making. Research aimed at further elucidating the molecular mechanisms and antigenic targets of different forms of myelitis is needed to improve treatments and long-term outcomes for children with myelitis.

## References

[1] Pidcock F.S., Krishnan C., Crawford T.O., Salorio C.F., Trovato M., Kerr D.A. (2007). Acute transverse myelitis in childhood: center-based analysis of 47 cases. Neurology.

[2] Absoud M., Greenberg B.M., Lim M., Lotze T., Thomas T., Deiva K. (2016). Pediatric transverse myelitis. Neurology.

[3] Abboud H., Petrak A., Mealy M., Sasidharan S., Siddique L., Levy M. (2016). Treatment of acute relapses in neuromyelitis optica: Steroids alone versus steroids plus plasma exchange. Mult. Scler..

[4] Thomas T., Branson H.M., Verhey L.H., Shroff M., Stephens D., Magalhaes S., Banwell B. (2012). The demographic, clinical, and magnetic resonance imaging (MRI) features of transverse myelitis in children. J. Child Neurol..

[5] Transverse Myelitis Consortium Working Group (2002). Proposed diagnostic criteria and nosology of acute transverse myelitis. Neurology.

[6] Goh C., Desmond P.M., Phal P.M. (2014). MRI in transverse myelitis. J. Magn. Reson. Imaging.

[7] Wang C., Narayan R., Greenberg B. (2018). Anti-myelin oligodendrocyte glycoprotein antibody associated with gray matter predominant transverse myelitis mimicking acute flaccid myelitis: A presentation of two cases. Pediatr. Neurol..

[8] Dubey D., Pittock S.J., Krecke K.N., Morris P.P., Sechi E., Zalewski N.L., Weinshenker B.G., Shosha E., Lucchinetti C.F., Fryer J.P. (2018). Clinical, radiologic, and prognostic features of myelitis associated with myelin oligodendrocyte glycoprotein autoantibody. JAMA Neurol..

[9] Virani S., Tan M., Abraham J. (2009). Transverse myelitis: Amphiphysin autoimmunity paraneoplastic syndrome in a woman with breast cancer. Clin. Adv. Hematol. Oncol..

[10] Keegan B.M., Pittock S.J., Lennon V.A. (2008). Autoimmune myelopathy associated with collapsin response-mediator protein-5 immunoglobulin G. Ann. Neurol..

[11] Flanagan E.P., Hinson S.R., Lennon V.A., Fang B., Aksamit A.J., Morris P.P., Basal E., Honorat J.A., Alfugham N.B., Linnoila J.J. (2017). Glial fibrillary acidic protein immunoglobulin G as biomarker of autoimmune astrocytopathy: Analysis of 102 patients. Ann. Neurol..

[12] Noland D.K., Greenberg B.M. (2018). Safety and efficacy of plasma exchange in pediatric transverse myelitis. Neurol. Clin. Pract..

[13] Barile L., Lavalle C. (1992). Transverse myelitis in systemic lupus erythematosus—The effect of IV pulse methylprednisolone and cyclophosphamide. J. Rheumatol..

[14] Zalewski N.L., Flanagan E.P., Keegan B.M. (2018). Evaluation of idiopathic transverse myelitis revealing specific myelopathy diagnoses. Neurology.

[15] Flanagan E.P., Weinshenker B.G., Krecke K.N., Lennon V.A., Lucchinetti C.F., McKeon A., Wingerchuk D.M., Shuster E.A., Jiao Y., Horta E.S. (2015). Short myelitis lesions in aquaporin-4-IgG-positive neuromyelitis optica spectrum disorders. JAMA Neurol..

[16] Wingerchuk D.M., Lennon V.A., Lucchinetti C.F., Pittock S.J., Weinshenker B.G. (2007). The spectrum of neuromyelitis optica. Lancet Neurol..

[17] Trebst C., Jarius S., Berthele A., Paul F., Schippling S., Wildemann B., Borisow N., Kleiter I., Aktas O., Kümpfel T. (2014). Update on the diagnosis and treatment of neuromyelitis optica: recommendations of the Neuromyelitis Optica Study Group (NEMOS). J. Neurol..

[18] Centers for Disease Control and Prevention AFM Investigation 2019. https://www.cdc.gov/acute-flaccid-myelitis/afm-surveillance.html.

[19] Fernandez-Garcia M.D., Kebe O., Fall A.D., Dia H., Diop O.M., Delpeyroux F., Ndiaye K. (2016). Enterovirus A71 genogroups C and E in children with acute flaccid paralysis, West Africa. Emerg. Infect. Dis..

[20] Martin J.A., Messacar K., Yang M.L., Maloney J.A., Lindwall J., Carry T., Kenyon P., Sillau S.H., Oleszek J., Tyler K.L. (2017). Outcomes of Colorado children with acute flaccid myelitis at 1 year. Neurology.

